# Ultrasensitive turn-off fluorescent sensor for estimation of the new influenza antiviral prodrug baloxavir marboxil in its pharmaceutical formulation

**DOI:** 10.1098/rsos.241634

**Published:** 2025-01-08

**Authors:** Amira S. Gouda, Mamdouh R. Rezk, Ahmed M. Abdel-Megied, Hoda M. Marzouk

**Affiliations:** ^1^ Zi diligence Biocenter, Bioequivalence Research, El-Mokattam, Cairo 11571, Egypt; ^2^ Pharmaceutical Analytical Chemistry Department, Faculty of Pharmacy, Cairo University, Cairo 11562, Egypt; ^3^ Pharmaceutical Analytical Chemistry Department, Faculty of Pharmacy, Kafr El-Sheikh University, Kafr El-Sheikh City 33511, Egypt; ^4^ Department of Pharmaceutical Sciences, Notre Dame of Maryland University, School of Pharmacy, Baltimore, MD 21210, USA

**Keywords:** baloxavir marboxil, content uniformity, fluorescent, nanosensors, nitrogen–sulfur–carbon quantum dots

## Abstract

Carbon quantum dots (CQDs) are a recently developed class of fluorescent nanoparticles made from carbon. Co-doping with heteroatoms such as nitrogen and sulfur improved the properties and generated a high quantum yield. In the proposed study, we utilized a simple, cost-effective, single-stage hydrothermal approach to produce extreme photoluminescence co-doped, nitrogen and sulfur, CQDs (N,S-CODs). Thiosemicarbazide was used as a nitrogen and sulfur source, while citric acid was used as a carbon source to produce fluorescent probes. The prepared N,S-CQDs were subjected to extensive characterization. The generated N,S-CQDs yielded strong fluorescence emission at *λ*_em_ 430.0 nm after excitation at *λ*_ex_ 360.0 nm, with a relatively high quantum yield of 41.3% utilizing quinine sulfate as a reference fluorescent compound. These N,S-CQDs were applied as fluorescent nanosensors for the ultrasensitive spectrofluorimetric determination of baloxavir marboxil (BXM) directly without pre-derivatization for the first time. BXM effectively quenches the native fluorescence of N,S-CQDs. Considering the optimal conditions, the fluorescence intensity reduction of N,S-CQDs exhibited a ‘turn-off’ response to BXM at concentrations of 10.0–100.0 ng ml^−1^, with detection limits of 1.88 ng ml^−1^ and quantitation limits of 5.69 ng ml^−1^, respectively. The proposed method determined BXM successfully in its tablet dosage form and further expanded to confirm the content uniformity of the tablet units in agreement with USP guidelines.

## 1. Introduction

Since their discovery in 2004, carbon quantum dots (CQDs) have grabbed the attention of researchers in different fields because of their excellent photo-physical properties, occurring in nano sizes ranging from 2 to 10 nm, easy synthesis methods, biodegradability, high solubility, affordability and eco-friendliness, as well as their strong fluorescence emission [1–8]. CQDs have been successfully applied across various fields, including photocatalysis, photovoltaics, light-emitting diodes (LEDs), nanotechnology, drug delivery, gene delivery, bioimaging and biosensing [9–14]. There have been publications on both top-down [15,16] and bottom-up [17] ways to synthesize CQDs [18]. The top-down technique allows the production of higher amounts of carbon dots. Nonetheless, it requires harsh experimental conditions (such as strong acids and arc discharges), expensive equipment and difficulties in controlling the size and morphology of the carbon dots. In contrast, bottom-up approaches use templates, and microwave and hydrothermal processes. The bottom-up approach is often used in the synthesis of carbon dots, which require precise control over their size and shape [19].

Various reported methods for synthesizing CQDs require costly equipment and sophisticated procedures. Moreover, they render insufficient quantum yield. In the present study, a single-stage hydrothermal approach was employed to create nitrogen and sulfur CQDs (N,S-CQDs). Citric acid (CA) and thiosemicarbazide (TSC) were employed as the source of carbon, nitrogen and sulfur, in order [20–23].

The incorporation of heteroatoms such as phosphorus, boron, nitrogen and sulfur into the structure of CQDs was performed through doping, whether surface modifications were made or not. Doping CQDs enhances their fluorescence and quantum yield, making them suitable for a variety of applications [24–28]. Nitrogen has a similar atomic radius to carbon, while the electronegativity is similar for sulfur and carbon, making nitrogen and sulfur CQDs an important doped type of CQD [29].

**Figure 1.**
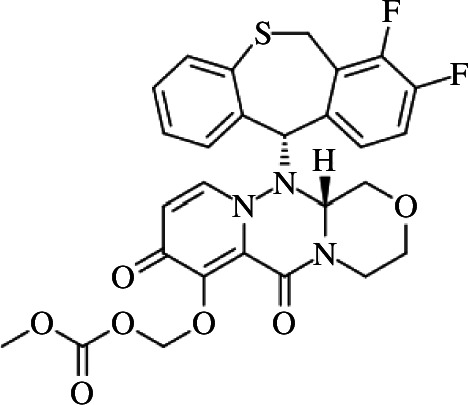
Chemical structure of baloxavir marboxil (BXM).

New antiviral medications have been developed recently for the treatment of different viral diseases, despite the presence of many vaccinations that have proved effective prevention of the development of the virus. Baloxavir marboxil (BXM; figure 1) [30] is one of the currently approved U.S. Food and Drug Administration (FDA) drugs for the treatment of influenza (flu) virus [31]. BXM is a prodrug that through hydrolysis gives baloxavir acid (BXA; [30]), the active form. BXA suppresses flu virus replication by targeting the cap-dependent endonuclease enzyme found in the viral RNA polymerase complex [31]. Since 2020 the world has been looking for a novel cure for SARS-Cov-2. Since flu and SARS-Cov-2 are both RNA viruses, BXA has potential effectiveness against SARS-Cov-2 correspondingly through blocking RNA synthesis. Some recent research has proven the *in vitro* antiviral effectiveness of BXA against SARS-Cov-2 [32].

Based on the authors’ knowledge and review of the literature, few analytical methods for estimating BXM in dosage forms and in human plasma have been reported [33–39]. However, no method has been published so far for the spectrofluorimetric determination of BXM using CQDs as fluorescent nanosensors. In this study, for the first time, a simple, sensitive and green spectrofluorimetric method was developed for the determination of BXM without the requirement for pre-derivatization, with good application in assaying as well as ensuring the content uniformity of the marketed tablets as a crucial step before starting bioequivalence studies. The proposed technique enhances the gradual quenching for the native fluorescence of N,S-CQDs with increasing drug concentrations.

Currently, one of the world’s main aims is to reduce or eradicate the risk of using certain hazardous chemical materials, acknowledging the value of adhering to green chemistry and the adoption of green chemistry concepts and techniques in analytical laboratories; we seeks to render the laboratory less harmful to the surroundings, which is a required goal for the majority of analysts [36,40–43]. The environmental impact and practical efficacy of the proposed method were evaluated using trustworthy and modern metric tools like Analytical Greenness (AGREE) and the new Blue Applicability Grade Index (BAGI) software. Finally, the Red–Green–Blue (RGB) 12 algorithm Excel sheet was used to promote the synergy between the analytical, environmental and practical aspects of the developed method, demonstrating the superiority of being greener and more potentially applicable compared with the reported high-performance liquid chromatography (HPLC) method [36].

## 2. Experimental

### 2.1. Instrumentation

Fluorescence intensities and spectra were reported using a Cary Eclipse^®^ fluorescence spectrophotometer (Agilent Technologies, USA). The excitation and emission monochromators had a slit width of 10 nm, a smoothing factor of 20 and an instrument voltage of 750 V. An Ohaus pH meter (Nänikon, Switzerland) was used for pH adjustment. UV–visible (UV–Vis) measurements were performed using a double-beam spectrophotometer (Taisite, USA). To record the Fourier transform infrared (FT-IR) spectra, a Nicolet-iS10 FT-IR spectrometer (Thermo Fisher, USA) was utilized. A total of 32 scans were recorded over a spectral range of 4000 to 400 cm^−1^ with a spectral resolution of 4 cm^−1^. The morphology of N,S-CQDs was examined by JEM−2100 high resolution transmission electron microscopy (HRTEM, JEOL, Japan). A Cu-grid covered with 200-mesh carbon was used for sample examination, with a working voltage of 200 kV (JEOL, Japan). In addition, elemental analysis of N,S-CQDs was conducted using an energy-dispersive X-ray (EDX) spectrometer attached to an electron microscope (JEOL, Japan).

### 2.2. Material and reagents

A baloxavir marboxil analytical standard with a purity of 99.10% was supplied by Siddhivinayak Chemicals (Mumbai, India). XOFLUZA^®^ tablets (Shionogi Pharma, Osaka, Japan) were obtained from a drugstore; each tablet contained 20 mg BXM.

TSC, CA, sodium hydroxide, potassium dihydrogen phosphate, dipotassium hydrogen phosphate, glacial acetic acid, boric acid, phosphoric acid, methanol and double-distilled water were purchased from Sigma-Aldrich (MO, USA). The universal buffer Britton–Robinson (BR) was prepared at a concentration of 0.02 M (pH 2–12), and phosphate buffer (0.05 M, pH 2–9) was freshly produced [44].

### 2.3. Standard solutions

BXM stock solution of 10.0 µg ml^−1^ was prepared by dissolving 0.01 g of BXM in methanol. The working solutions were produced by diluting aliquots of the stock solution in distilled water and stored in a calibrated refrigerator at a temperature of 5 ± 3°C for two weeks.

### 2.4. Synthesis of nitrogen,sulfur carbon quantum dots

The co-doped nitrogen and sulfur carbon quantum dots (N,S-CQDs) were produced hydrothermally using CA and TSC [20,21]. About 0.68 g of TSC was mixed with 0.52 g of CA, dissolved in 20.0 ml of distilled water, sonicated for 15 min and refluxed at a temperature of 160°C for 12 h until a dark orange colour was obtained, indicating the synthesis of the fluorescent N,S-CQDs. The fluorescent solution was then allowed to cool and kept in the refrigerator to be used throughout the study.

### 2.5. Spectrofluorimetric measurements

To optimize all factors influencing the fluorescence detection of the examined drug, 100.0 ml of N,S-CQDs were mixed with 200.0 ng ml^−1^ of BXM solution. The intensity of emitted fluorescence was measured at 430.0 nm directly after excitation at 360.0 nm. To serial concentrations of BXM, a quantity of 100.0 µl of N,S-CQDs was added and incubated at 25°C for 10 min. Calibration curves were constructed by drawing a graph with the drug concentration in ng ml^−1^ against the fluorescence intensity reduction. The regression equations were further constructed.

### 2.6. Analysis of baloxavir marboxil in XOFLUZA^®^ tablets

Ten XOFLUZA^®^ tablets were powdered and mixed. An accurately weighed amount of the powder equivalent to 20 mg of BXM was transferred into a conical flask containing 50 ml of methanol, sonicated for 10 min, filtered into a 100 ml measuring flask and diluted to volume with methanol. Further dilutions were performed, and the volume was made up with distilled water. The concentration of BXM in tablets was quantified using a calculated regression equation. A standard addition approach was used with various ratios of the pure standard of BXM and well known quantities of the pharmaceutical product, and the results were handled as previously described under §2.5.

### 2.7. Content uniformity testing of tablets

Following the United States Pharmacopeial guidelines [45], to assess the content uniformity of BXM tablets [46], analysis was conducted as previously described for determining BXM in XOFLUZA^®^ tablets except that 10 individual tablets were used separately. Appropriate dilutions were carried out using distilled water in order to obtain a final concentration of 40 ng ml^−1^.

### 2.8. Quantum yield measurements

Determination of the quantum yield of the created dots was performed through the single-point approach. The following equation was used for the calculation of N,S-CQDs fluorescence quantum yield [21,47]:


(2.1) $$\Phi_{N,S-CQDs}=\Phi_{QS}\times (F_{N,S-CQDs}/F_{QS})\times (A_{QS}/A_{N,S-CQDs})\times (\eta_{N,S-CQDs}/\eta_{QS}{)}^{2},$$

where *Փ* represents the quantum yield, *F* reflects the combined fluorescence emissions intensity, *A* refers to the absorbance value and *η* represents the distilled water refractive index. Quinine sulfate (QS) was the standard fluorophore used at 350.0 nm giving a quantum yield of 0.54 in 0.1 M H_2_SO_4_. For aqueous solvents, *η*_N,S-CQDs_/*η*_QS_ corresponds to 1.

## 3. Results and discussion

In this study, a sensitive spectrofluorimetric approach for detecting BXM in its tablet dosage form was performed using quenching of the synthesized blue fluorescent N,S-CQDs by the drug.

### 3.1. Synthesis of carbon quantum dots

The study utilized a straightforward technique for synthesizing strongly fluorescent N,S-CQDs. The strategy involved a single-stage hydrothermal approach to create N,S-CQDs. CA and TSC were employed as the source of carbon, and nitrogen and sulfur, respectively [20]. The process gave a relatively high quantum yield of 41.3%. A marked blue fluorescence was observed for the N,S-CQDs under UV light. The N,S-CQDs remained homogeneous and did not precipitate for a duration of two weeks stored in a sealed container in the refrigerator.

### 3.2. Characterization of nitrogen,sulfur carbon quantum dots

#### 3.2.1. High-resolution transmission electron microscopy

**Figure 2.**
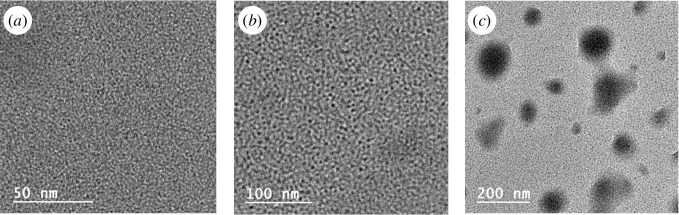
High-resolution transmission electron microscopy images of the prepared N,S-CQDS at different magnifications. Scale bars: (*a*) 50 nm, (*b*) 100 nm, and (*c*) 200 nm.

HRTEM was utilized to examine the shape and surface morphological properties of the N,S-CQDs fluorescent probe in nanoscale. The samples were placed on a carbon-coated Cu-grid (200 mesh) and analysed using HRTEM at a voltage of 200 kV [48,49]. As shown in figure 2, N,S-CQDs have a well dispersed spherical form without any noticeable aggregation, with particle diameters distributed in the range of 8–20 nm, confirming the size of the particles to be in the nano-scale.

#### 3.2.2. Ultraviolet–visible and fluorometric investigation

**Figure 3.**
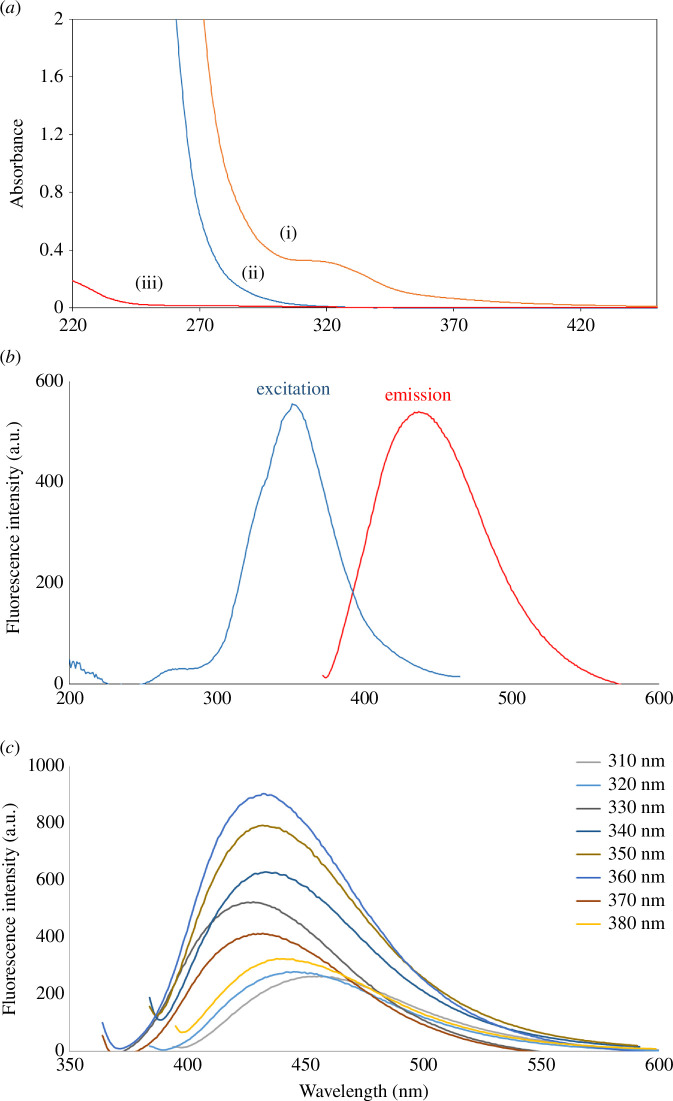
(*a*) UV–visible absorption spectra of N,S-CQDS (i), TSC (ii) and CA (iii). (*b*) Fluorescence emission spectrum of N,S-CQDs at 430.0 nm after excitation at 360.0 nm. (*c*) Fluorescence spectra of N,S-CQDs at different excitation wavelengths.

Figure 3*a* presents the UV spectra of N,S-CQDs, CA and TSC. N,S-CQDs exhibited a UV absorption band at 320 nm [20,50,51]. Figure 3*b* displays the fluorescence emission and excitation spectra of N,S-CQDs in aqueous solvent. The optimal excitation and emission wavelengths were *λ*_ex_ = 360.0 and *λ*_em_ = 430.0 nm, respectively. The excitation wavelength varied from 310.0 to 380.0 nm; consequently, the fluorescence spectra of N,S-CQDs shifted, with the highest fluorescence intensity observed at 360.0 nm, as shown in figure 3*c*.

#### 3.2.3. Energy-dispersive X-ray spectroscopy

**Figure 4.**
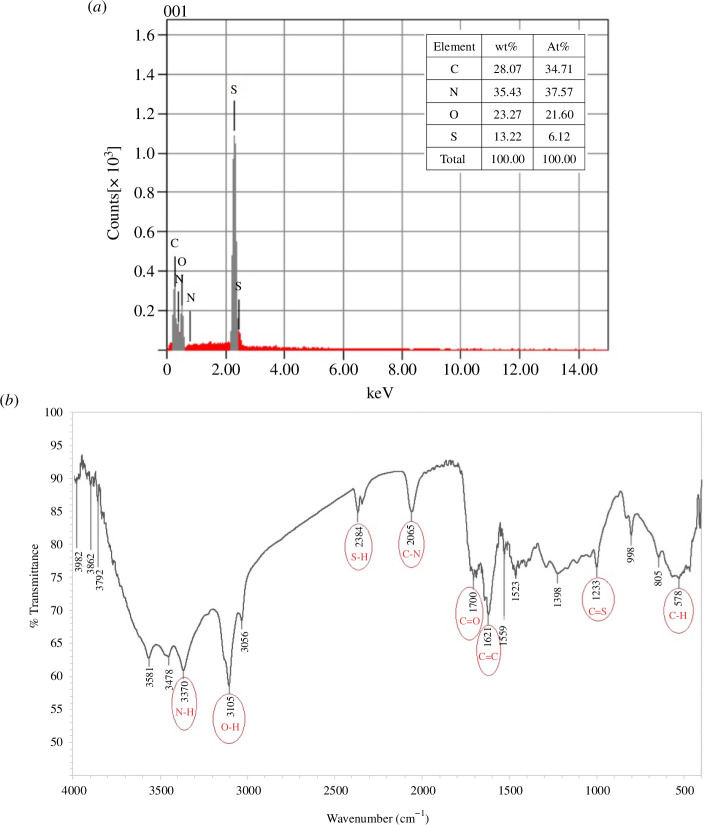
(*a*) Energy-dispersive X-ray spectrum of synthesized N,S-CQDs and (*b*) Fourier transform infrared spectrum presenting the surface functionality of N,S-CQDs.

To investigate the elemental composition of the quantum dots and the degree of nitrogen doping, EDX spectroscopy was used, which confirmed they were mostly composed of carbon, oxygen and nitrogen. Furthermore, it was shown that an enhanced nitrogen doping level was attained [27,52,53], as illustrated in figure 4*a*.

#### 3.2.4. Fourier transform infrared spectroscopy

The primary surface functional groups of N,S-CQDs were investigated via FT-IR analysis. The FT-IR spectrum, displayed in figure 4*b*, shows multiple N–H/O–H stretching vibrations where the broad bands are in the region 3500–3100 cm^−1^. The existence of the O–H group was confirmed by a peak at 3105 cm^−1^, while the N–H group appeared at 3370 cm^−1^. The peak at 2065 cm^−1^ is due to the C–N group, while the band at 2371 cm^−1^ could be due to the –SH group. The C=O of the carboxylic acid group is visible at 1700 cm^−1^, and the stretching maxima of 1233, 1621 and 578 cm^−1^ are a result of C=S, C=C and C–H bonds, respectively [20,54–56]. This confirms the water solubility of the prepared CQDs owing to the presence of hydroxyl and carbonyl groups.

### 3.3. Quenching mechanism

**Figure 5.**
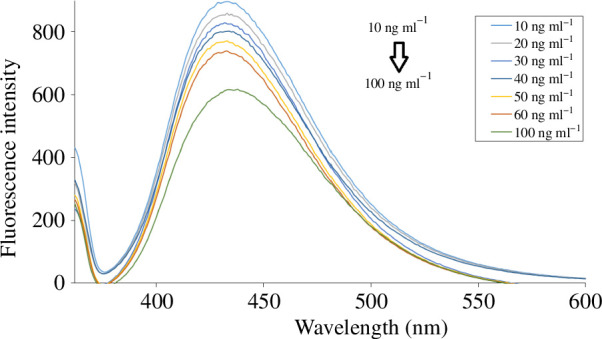
Fluorescence emission spectra of N,S-CQDs in aqueous solution upon addition of various concentrations of BXM (from top to bottom: 0, 10.0, 20.0, 30.0, 40.0, 50.0, 60.0 and 100.0 ng ml^−1^).

Fluorescence quenching can occur through several mechanisms, including static quenching, dynamic quenching and the inner filter effect (IFE; [57]). On spiking of the synthesized N,S-CQDs solution with aqueous solutions of BXM, the CQDs' native fluorescence was quantitatively quenched. It was noticed that upon increasing the concentration of BXM the synthesized dots’ fluorescence spectrum decreased accordingly, as shown in figure 5. In this study, an overlap was noticed between the absorbance spectrum of BXM and the generated quantum dots' excitation spectrum, as presented in electronic supplementary material, figure S1; IFE could be the quenching mechanism. The fluorescence intensity of N,S-CQDs was corrected for potential inner filter effects (IFE) caused by light absorption and scattering with increasing concentrations of BXM utilizing equation (3.1) [58]:


(3.1) $$F_{corr}=F_{obs}\times 10^{(A_{ex}+A_{em})/2},$$

where *F*_corr_ and *F*_obs_ are the corrected fluorescence and observed fluorescence, and *A*_ex_ and *A*_em_ represent the absorbance of the drug solution at *λ*_ex_ = 360.0 and *λ*_em_ = 430.0 nm, respectively.

The quenching efficiency (%*E*) for either the corrected or observed fluorescence, was determined via equation (3.2) [58]:


(3.2) $$\%E=\left(1-\left(F/F_{0}\right)\right)\times 100,$$

where %*E* is the suppressed efficiency, while *F* corresponds to either *F*_corr_ or *F*_obs_, and *F*_0_ is the blank fluorescence intensity. The values of %*E* of the corrected and observed intensities were plotted individually against BXM concentrations in ng ml^−1^. Electronic supplementary material, figure S2A shows that IFE has a substantial influence in the N,S-CQDs quenching. This is due to BXM having a strong absorbance at 360.0 nm.

Along with IFE, various mechanisms might occur. The Stern–Volmer equation (3.3) [58] revealed potential mechanisms that could explain the quenching of N,S-CQDs’ natural fluorescence:


(3.3) $$F_{0}/F=1+K_{SV}[Q]=1+K_{q}\tau 0[Q].$$

The fluorescence intensities in the absence and presence of a quencher are represented by *F*_0_ and *F*, respectively. The bimolecular quenching rate constant (*K*_q_), and the Stern–Volmer quenching constant (*K*_SV_) are also mentioned. (τ0) refers to the average lifetime (10^-8^s) and the concentration of quencher is represented by [Q] [59].

Static and dynamic quenching can be distinguished by their temperature dependence. Increasing temperature typically reduces the likelihood of complex formation in static quenching, leading to a decrease in the Stern–Volmer quenching constant *K*_SV_. On the other hand, dynamic quenching is more likely to occur at higher temperatures owing to increased molecular motion, resulting in an increase in *K*_SV_. In our study, Stern–Volmer plots demonstrated that *K*_SV_ values dropped with increasing temperature (298, 308 and 318 K), supporting the static quenching mechanism (electronic supplementary material, tables S1 and figure S2B). The study concluded that the mechanisms for quenching the fluorescence intensity of the N,S-CQDs when exposed to the drug under study were both the IFE and static quenching.

### 3.4. Optimization of factors affecting the fluorescence sensing of baloxavir marboxil

#### 3.4.1. Effect of pH

To thoroughly investigate the impact of pH on the fluorescence intensity of N,S-CQDs quenched by BXM, various buffers (acetate, phosphate and borate) were tested across a pH range of 3–11. The results showed no significant variation in fluorescence intensity across the different pH levels. This finding suggests that the method can be performed under neutral conditions, eliminating the need for pH adjustments in future experiments.

#### 3.4.2. Effect of temperature

The effect of temperature was examined within the range 25−60°C. It was observed that fluorescence quenching decreased progressively as the temperature increased (electronic supplementary material, figure S3A). Therefore, the study was conducted at room temperature.

#### 3.4.3. Effect of incubation time

To study the incubation duration of N,S-CQDs with BXM, emission fluorescence spectra were recorded at intervals of 1 min to 1 h. After combining the drug and QDs, the results showed a rapid increase that plateaued after 10 min, as shown in electronic supplementary material, figure S3B.

### 3.5. Assessment of the suggested method's sustainability

It is now strongly advised to use environmentally friendly solvents throughout the analytical process, from sample preparation to final sample determination. Several approaches were used to assess the greenness of the suggested method [40,60,61].

#### 3.5.1. Greenness assessment via Analytical Greenness metric tool

**Figure 6.**
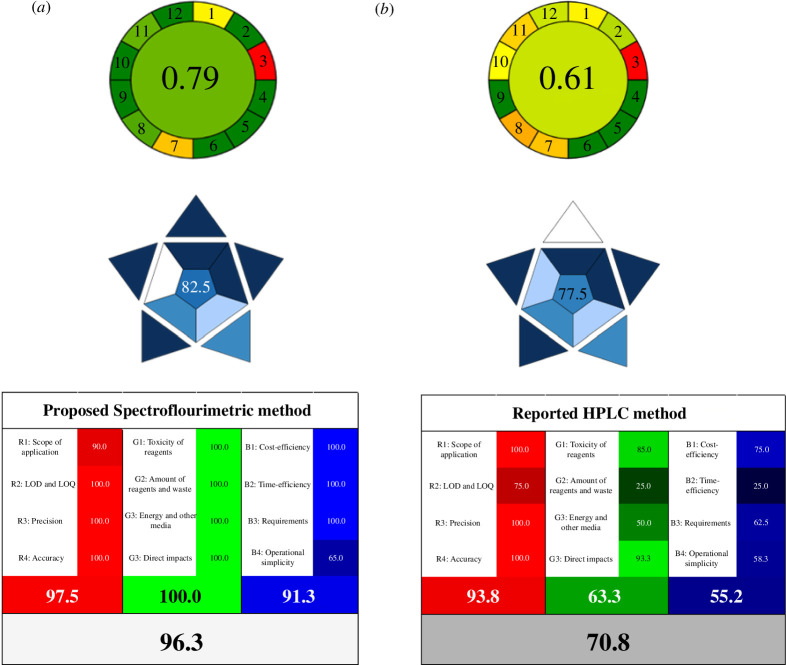
Greenness, blueness and whiteness assessment of (*a*) the proposed spectrofluorimetric and (*b*) the reported high-performance liquid chromatography (HPLC) [36] methods via AGREE, BAGI and RGB 12 whiteness tools. R, red; G, green; B, blue; LOD, limit of detection; LOQ, limit of quantification.

The sustainability of the proposed method was evaluated using the innovative free AGREE software [62], which evaluates the adherence of the analytical method to each of 12 distinct aspects of green analytical chemistry, coloured in a spectrum from dark green to red, creating a 12 segment circular pictogram. A final quantitative score was assigned on a scale of 0–1, with the closer the score to 1, the better the impact [63,64]. In comparison with the reported HPLC (score 0.61) procedure, the suggested spectrofluorimetric approach had a higher overall score (0.79), as shown in figure 6. Distilled water is considered the greenest and safest solvent to use during analysis. As a result, the spectrofluorimetric measurement of BXM using CQDs as fluorescent nanosensors could be accomplished without negatively impacting the environment.

#### 3.5.2. Assessment of the applicability of the proposed method according to the Blue Applicability Grade Index tool

To assess the method applicability, BAGI [65], which is a recently proposed user-friendly tool, was used. This tool emphasizes the practical fundamental components of the White Analytical Chemistry (WAC). BAGI uses simple free-download software to evaluate the analytical technique in 10 features. The result of the calculation is displayed as an asteroid pictogram divided into 10 sections, with a score in the centre [66]. Based on this new method, the final score must be higher than 60 to qualify the analysis process as practical and applicable. Both methods were evaluated using the BAGI tool, as shown in figure 6, which demonstrates the functionality and practicality of our suggested method, with higher score along with more dark blue sections when compared with the reported one.

#### 3.5.3. Whiteness assessment using RGB 12 algorithm tool

Sustainable development requires considering the wellbeing of the environment and the community. The RGB paradigm is effective for evaluating analytical methods globally. An analytical method’s efficiency in promoting sustainability is measured quantitatively [67,68].

This colour model is commonly used in electronics and expands the definition of ‘green chemistry’ to encompass other main colours. Analytical performance (as measured by validation criteria such as accuracy, precision, sensitivity and limit of detection (LOD)) is assigned red, while green compliance with Green Analytical Chemistry (GAC) environmental safety principles is achieved by reducing reagent toxicity and usage, minimizing waste generation, optimizing energy consumption and reducing overall environmental impact. Productivity and practical/economic performance (as measured by expense and minimum practical requirements) are assigned blue. To evaluate the method using the RGB 12 algorithm, the red, green and blue tables in the freely available Excel sheet template are filled out [68].

A computational assessment assigns a colour to the technique depending on the ratio of the three fundamental colours employed [69]. White represents the most perfect, sustainable and reliable analytical procedure. The template provides a detailed assessment, with scores ranging from 0 (worst probable outcome) to 100 (ideal for the intended application of the concept). Figure 6 shows that our proposed method has a higher whiteness score (96.3%) compared with the reported method (70.8%), indicating greater sustainability and efficiency.

### 3.6. Method validation

The proposed method was validated based on the International Council on Harmonisation Q2(R1) recommendations [70]. The calibration curves were created by plotting drug concentrations in ng ml^−1^ against the fluorescence quenching ( $F_{0}-F$ ). Table 1 demonstrates that the drug was linear across all concentration levels, with an optimal correlation coefficient of 0.9994. The linear regression is expressed as follows:


(3.4) $$F_{0}-F=3.0729C+43.613\;(r=0.9994),$$

**Table 1.** Analytical performance data and validation parameters of the proposed method for determination of baloxavirmarboxil (BXM) in pure form. RSD, relative standard deviation; LOD, limit of detection; LOQ, limit of quantitation.

method parameter	BXM
range (ng ml^−1^)	10.00–100.00
regression equation parameters slope (*b*)^a^ intercept (*a*)^a^ standard deviation of slope standard deviation of intercept correlation coefficient (*r*)	3.0717 43.6139 ±0.0335 ±1.7482 0.9994
accuracy (mean ± SD)^b^	100.16 ± 0.59
precision (±%RSD)^c^ (±%RSD)^d^	0.49 0.52
LOD (ng ml^−1^)^e^	1.88
LOQ (ng ml^−1^)^e^	5.69

^a^ Regression equation: *A = a + bc*, where *A* is the difference in fluorescence intensity (Δ*F* = *F* − *F*_0_), *c* is the concentration (ng ml^−1^), *b* is the slope and *a* is the intercept.

^b^ Accuracy (average of three different concentrations (24.0, 32.0 and 48.0 ng ml^−1^) of three replicates each).

^c^ Intra-day precision (average of three different concentrations (24.0, 32.0 and 48.0 ng ml^−1^) of three replicates each, within the same day).

^d^ Inter-day precision (average of three different concentrations (24.0, 32.0 and 48.0 ng ml^−1^) of three replicates each, repeated on three successive days).

^e^ LOD and LOQ are calculated according to the International Council on Harmonisation guideline (ICH), 3.3 × s.d. of *y*-intercept/slope and 10 × s.d. of *y*-intercept/slope, respectively.

where *F* is the intensity of the fluorescence in the presence of BXM, while *F*_0_ is the intensity of N,S-CQDs’ native fluorescence without the drug, *C* is the concentration of the drug in ng ml^−1^ and *r* is the correlation coefficient.

The accuracy of the devised method was evaluated by calculating the percentage recovery obtained by analysing three concentration levels across the linearity range (table 1). The inter-day and intra-day precision was evaluated using three replicates from three different concentrations of BXM examined on the same day or on three successive days. Results were accepted that showed a relative standard deviation (RSD) of <2% (table 1).

The results for limit of quantitation (LOQ) and LOD demonstrated the sensitivity of the proposed method and showed the method’s capacity to quantify the analyte in dosage forms, as summarized in table 1.

The suggested approach’s robustness was assessed by analysing the impact of deliberate experimental variation on fluorescence sensing, including reagent volume, and incubation duration. Electronic supplementary material, table S2 shows that minor experimental parameters did not significantly affect fluorescence intensity quenching.

The proposed method was able to selectively analyse the drug in the tablet dosage form with a high recovery rate (100.01%) and RSD values below 2%, indicating no influence from common excipients, as shown in table 2.

**Table 2.** Quantitative determination of baloxavir marboxil (BXM) in XOFLUZA^®^ tablets and application of standard addition technique.

%Found ± SD^a^	Standard BXM Addition Technique			
	Claimed (ng/mL)	Pure added (ng/mL)	Pure Found (ng/mL)	%Recovery of the pure added^b^
100.01 ± 1.16	15	15.00	14.87	99.13
		35.00	35.13	100.37
		55.00	53.94	98.07
	Mean ± SD			99.19 ± 1.15

^a^ Average of six determinations.

^b^ Average of three determinations.

### 3.7. Method application

#### 3.7.1. Analysis of baloxavir marboxil in tablets

The suggested approach successfully quantified the BXM in the commercial dosage form XOFLUZA^®^ tablets. Drug concentrations were calculated using derived regression equations. Table 2 reveals an acceptable average percentage recovery (100.01%) for BXM in the dosage form. Additionally, a standard addition approach was successfully used to ensure the correctness of the process (table 2).

The established method was found to be accurate and precise, as evidenced by good agreement between the results and those from the comparison method [36] (electronic supplementary material, table S3).

Moreover, compared with the previously reported spectrofluorimetric method that used acetoxymercuric fluorescein as a quenching reagent [39], the proposed spectrofluorimetric method proved to be sensitive, fast, straightforward and cost-effective. The current work also provides the first published method for the spectrofluorimetric determination of BXM using CQDs as fluorescent nanosensors, enabling efficient determination of BXM in pharmaceutical preparations. It also surpasses previous methods in eco-friendliness, efficiency, cost-effectiveness and applicability for assaying BXM in tablet dosage forms, as demonstrated in electronic supplementary material, table S4. Accordingly, these results highlight how quantum dots, recognized for their unique optical properties and strong fluorescence, offer significant potential in analytical applications by enhancing sensitivity and broadening detection capabilities [71–73].

#### 3.7.2. Content uniformity testing of baloxavir marboxil tablets

The suggested technique accurately measured the fluorescence intensity of extracts of individual tablets, allowing efficient evaluation of the content uniformity of the BXM in accordance with US Pharmacopeia (USP) requirements [45]. Table 3 shows that BXM has high content uniformity since the computed acceptance value (AV) is much lower than the maximum permissible acceptance value (L1).

**Table 3.** Results of content uniformity testing for determining baloxavir marboxil in XOFLUZA^®^ tablets by the proposed method. RSD, relative standard deviation; AV, acceptance value.

	tablet no.	% of label claim
	1	98.56
	2	99.68
	3	97.96
	4	98.22
	5	100.27
	6	98.74
	7	99.50
	8	98.52
	9	100.78
	10	98.45
mean		99.07
s.d.		0.94
%RSD		0.95
AV		2.26
maximum allowed AV (L1)		15.0

## 4. Conclusion

This work presents a facile, straightforward, low-cost and green method for determining BXM in its dosage form by employing N,S-CQDs as fluorescent probes for drug detection. A single-stage hydrothermal approach was used to create N,S-CQDs utilizing the simple precursors TSC and CA with a relatively high quantum yield of 41.3%. The fluorescence of the N,S-CQDs was obviously reduced as a result of the static quenching mechanism and a complementary IFE. The proposed technique was validated using the International Council on Harmonisation Q2(R1) standards. The developed spectrofluorimetric method demonstrated high selectivity in assaying the pharmaceutical dosage form as well as content uniformity testing by monitoring the quantitative fluorescence quenching of N,S-CQDs at increasing concentrations. Moreover, sustainability of the proposed method was appraised via the AGREE, BAGI and WAC tools, which confirmed its superiority compared with the traditional HPLC method.

## Data Availability

All data generated or analysed during this study are included in this published article. Supplementary material is available online [74].
